# Application of Antisolvent Precipitation Method for Formulating Excipient-Free Nanoparticles of Psychotropic Drugs

**DOI:** 10.3390/pharmaceutics14040819

**Published:** 2022-04-08

**Authors:** Carina Yeeka Wu, Wei Wang

**Affiliations:** Center for Pharmacy & Department of Chemistry, University of Bergen, 5020 Bergen, Norway; carina.wu@student.uib.no

**Keywords:** antisolvent, precipitation, nanodrug, suspension, supersaturation

## Abstract

The aim of the present study was to systematically examine the effects of variations in the process parameters of the antisolvent precipitation method employed in the preparation of excipient-free pure nanoparticles of five existing/potential psychotropic drugs, namely amitriptyline hydrochloride (AMI), coumarin 6 (COU), curcumin (CUR), nortriptyline hydrochloride (NOR), and prochlorperazine dimaleate (PRO). In the preparation protocols employed, AMI and NOR were expected to be charged enough to be identified as surface-active molecules. Through the employment of five different preparation protocols, the effects of varying the flow rate, the compound concentration in the solvent solution $C_{0}^{solvent}$, the solvent:antisolvent ratio (SAS-ratio), and pH of the antisolvent on the final size of the particles $D_{H}^{f}$ were investigated in detail and the results were explained using available theories for the antisolvent precipitation method. We found that $D_{H}^{f}$ increased with the average of the octanol-water partition coefficients (log*P*)*_av_* of the compound. Moreover, the average of the final particle sizes ${\left(D_{H}^{f}\right)}_{av}$ increased linearly with (log*P*)*_av_*. These findings are useful for predicting the size of nanodrugs prepared through the antisolvent precipitation method.

## 1. Introduction

Psychiatric disorders represent an increasing health burden on an international level and are often associated with a great socio-economical and emotional burden for patients and caregivers. Worldwide, around 450 million people have experienced suffering due to psychiatric disorders, with depression (300 million), bipolar affective disorder (30 million), and schizophrenia (23 million) being the most common conditions [1]. According to the World Health Organization (WHO), mental health problems will be the main cause of mortality and morbidity by 2030. One of the major problems regarding psychiatric patients is their significant non-adherence to the treatment [2]. While some patients are incapable of taking their medication, others choose to refrain from the treatment, possibly because of the experience of the side effects [2], lack of therapeutic effects, or because of a delay in the initial response [3].

By downsizing the drug particles, a much larger effective surface area could be achieved and the accompanied increased dissolution rate and saturated solubility may improve the bioavailability of drugs [4,5]. The blood–brain barrier (BBB) is the most important obstacle encountered during drug delivery to the brain [6]. The BBB hinders many psychotropic drugs from obtaining therapeutic levels in the brain without high levels of drugs in the systemic circulation, which encourages unwanted side effects (e.g., gastrointestinal side effects), e.g., by the intrathecal route [7]. Nanoparticles could be delivered once and serve as a long-lasting depot in the brain. They can be easily radiolabeled, so it is possible to precisely deliver the desired dose to the right part of the brain using the intra-arterial route [8] and overcome compliance issues of patients with mental illnesses. The intra-arterial route is not the only one to go forward as the focused ultrasound of intranasally delivered particles is another important approach [9]. Focused ultrasound can also improve the interstitial dispersion of stereotactically injected nanoparticles [10].

Among all of the nanoparticulate drug delivery systems, those based on polymers and lipids (i.e., liposomes) represent a significant portion of the drug delivery systems that reach clinical trials [11]. However, for such systems, a substantial number of additives are required and the techniques applied for their preparation have a time-consuming nature [12,13]. Thus, in the present study, nanoparticles were synthesized in the absence of excipients. Due to their high mass per volume loading, dense and solid-state drugs offer many advantages, especially in situations where high dosing is required [14]. Furthermore, high loading is also crucial for low-volume administration routes, e.g., in intramuscular and ophthalmic applications [13,15].

The preparation of nanoparticles in the absence of excipients can be broadly classified into two approaches, namely top-down and bottom-up. The top-down approach focuses on the size-reduction of bigger particles to nanosized particles (i.e., milling). Essentially, every drug can be milled to smaller particles regardless of their solubility in aqueous or non-aqueous solvents [16]. It should be added that top-down techniques are identified as time-consuming, high-energy methods with low particle size uniformity and potential contamination challenges [17,18].

In the bottom-up approach, nanoparticles are generated from drug molecules in solution using, e.g., the antisolvent precipitation method or evaporative precipitation technique [19,20]. This approach uses simple instruments that require a low mechanical energy input during the whole process of dissolution and precipitation/drying [21]. Moreover, in the bottom-up approach, a narrower size distribution can be achieved (compared with the top-down approach) and the manufacturing process can be operated at a low temperature, making it suitable for thermolabile drugs [22]. It should be added that the quick arrangement of molecules during the precipitation can lead to substantial amorphousness [17,22].

Thus, in the present study, we employed one of the methods following the bottom-up approach, namely the antisolvent precipitation method. Nanoparticles were created for a number of potential and existing psychotropic drugs, namely curcumin (CUR), coumarin 6 (COU), nortriptyline hydrochloride (NOR), amitriptyline hydrochloride (AMI), and prochlorperazine dimaleate (PRO). AMI and NOR are well-known antidepressants. PRO is used in the symptomatic management of psychotic disorders and short-term management of nonpsychotic anxiety, while CUR exhibits significant antidepressant effects, possibly through the inhibition of monoamine oxidase A and B enzymes [3], and some coumarin derivatives are considered as potential atypical antipsychotics [23,24]. Throughout our experiments, we conducted a systematic study whereby the effects of changes in the flow rate, the compound concentration in the solvent solution $C_{0}^{solvent}$, the solvent:antisolvent volume ratio (SAS-ratio), and pH of the antisolvent on the size of the particles were elucidated. In addition, for the obtained particles, we characterized the morphology, confirmed the crystalline structure, and determined the surface charge using scanning electron microscopy (SEM), X-ray powder diffraction (XRD), and zeta potential measurements, respectively.

Analysis of the final size of the particles $D_{H}^{f}$ revealed that $D_{H}^{f}$ increased with the average of the octanol-water partition coefficients ${\left(\log P\right)}_{av}$ of the compound. Furthermore, the average of the final particle sizes ${\left(D_{H}^{f}\right)}_{av}$ increased linearly with ${\left(\log P\right)}_{av}$. To our knowledge, this is the first time that such a relationship between the particle size and logP has been identified. Moreover, throughout our experiments, AMI and NOR were expected to be charged enough to be identified as surface-active molecules [25]. It is of note that, to our knowledge, this is the first time that the antisolvent precipitation method has been employed for surface-active drugs.

## 2. Theory of Antisolvent Precipitation Methods

The principle behind the antisolvent precipitation method is to exploit the solubility of a drug in different miscible solvents, one good solvent and one bad solvent (antisolvent). First, the drug is dissolved in the solvent and, afterwards, is quickly mixed with the antisolvent. The fast diffusion of the drug solvent solution into the antisolvent causes a high supersaturation, which is the driving force for the precipitation [19]. The degree of supersaturation β can be defined as the ratio between the compound concentration in the solution $C_{0}$ (i.e., in the solvent + antisolvent mixture solution) and the compound solubility at the given condition $C^{*}$ [17,22,26,27]:

(1) $$\beta =\frac{C_{0}}{C^{*}}$$

The precipitation process consists of several steps: (i) nucleation, (ii) particle growth, and (iii) agglomeration. Classical nucleation theory describes the initial step of the crystallization as the spontaneous assembly of a few molecules, which creates a thin interface between a solid and a liquid phase [28]. Energy fluctuation in supersaturated solutions induces the coalescence of randomly diffused molecules, thus creating aggregates of several molecules that are called embryos. If the energy released during the formation of an embryo is higher than the energy required to stabilize the new surface, the embryos are converted into stable nuclei [22]. Thus, a critical energy barrier $\Delta G^{*}$ should be overcome for the nucleation process, which is related to the critical radius $r^{*}$. These can be described as [27].

(2) $$\Delta G^{*}=\frac{16\pi \gamma^{3}\Omega^{2}}{3k_{B}^{2}T^{2}{\left(\ln\beta \right)}^{2}}$$

(3) $$r^{*}=\frac{2\Omega \gamma }{k_{B}T\ln\beta }$$

where γ is the interfacial tension between the solid nucleus and the solution, Ω is the volume of a molecule inside the nucleus, T is the temperature (in kelvin), and $k_{B}$ is the Boltzmann constant.

The kinetic parameter describing the rate of nucleation J is affected by a prefactor ($A_{n}$), which is determined from kinetic considerations [22]:

(4) $$J=A_{n}\exp\left[\frac{-16\pi \gamma^{3}\Omega^{2}}{3k_{B}^{3}T^{3}{\left(\ln\beta \right)}^{2}}\right]$$

The parameters $\Delta G^{*}$, $r^{*}$ and J are all dependent on β. Any factor that increases $C_{0}$ (e.g., through an increase in $C_{0}^{solvent}$) or reduces $C^{*}$ (e.g., through increasing the amount of antisolvent or lowering the temperature) will increase β. According to Equations (2) and (4), a higher β and a lower γ lead to a lower $\Delta G^{*}$ and a higher J. Thus, since nucleation becomes preferred over particle growth, smaller particles are generated [22]. Increasing β results in a decrease in the particle size to a certain value [29]. A further increase favors a larger particle size through a higher availability of the solute molecules close to the growing surface.

After the formation of stable nuclei, particle growth happens through the condensation of molecules onto an already growing surface, thus reducing the supersaturation in the system [22]. The particles will continue to grow until $C_{0}$ reaches the equilibrium concentration or $C^{*}$. Growth beyond this arises due to aggregation or agglomeration, where particles collide and create larger particles, following the Smoluchowski kinetics rate:

(5) $$r_{s}=k_{s}n_{p}^{2}$$

where the particle collision rate $r_{s}$ is dependent on the rate constant $k_{s}$, calculated from the temperature and kinematic viscosity of the medium, and the number of particles per unit volume $n_{p}$.

During agglomeration and aggregation, the number of particles decreases and the particle size increases. The driving force behind all three stages of the precipitation process is supersaturation. Alteration of the parameters affecting supersaturation can therefore be used to design particles with desirable sizes. After particles are made, several destabilization routes, such as Ostwald ripening, may lead to further particle growth. Immediate drying, e.g., spray drying or freeze-drying, may provide prolonged stabilization [30].

## 3. Materials and Methods

### 3.1. Materials

Curcumin was received as a gift from Capital Medical University in China. Coumarin 6 (powder, 98%), nortriptyline hydrochloride (powder, ≥98%), amitriptyline hydrochloride (powder, 98%), prochlorperazine dimaleate (powder), and sodium hydroxide (pellets, ≥98%) were purchased from Sigma-Aldrich, Steinheim, Germany. Absolute ethanol (≥99%, Antibac, Asker, Norway), acetone (>99.5%, Sigma-Aldrich), and distilled water were used in the experiments. Standard buffer solutions of pH = 4.008, pH = 7.413, and pH = 11.00 were purchased from Riedel-de Haën, Steinheim, Germany.

### 3.2. Preparation Procedure

Solvent solutions with saturation concentration (Cs) and half of the saturation concentration (½Cs) of CUR, COU, NOR, AMI, and PRO were prepared and subsequently filtered through a 0.2-µm nylon syringe filter (Pall Corporation, Washington, DC, USA). The solvent solution of the compound (10 mL) was added to the antisolvent (100–200 mL) in portions of 1 mL at a fixed flow rate (1 or 2 mL/min) under the stirring speed of 1000 rpm (IKA RTC digital). A sample was removed after each addition. The samples were centrifuged at 20 °C and 3000 rpm (Hettich Universal 320R centrifuge, Bremen, Germany). The dynamic light scattering (DLS) data presented correspond to samples that were prepared by the re-dispersion of the centrifuged particles in antisolvent solutions, where a volume (950 µL) of the upper layer was removed for analysis.

The concentration of the compound in the solvent solution ($C_{0}^{solvent}$), centrifugation time, and used solvents and antisolvents, together with the details of the preparation protocols applied, are given in Table 1. The end dispersions were freeze-dried (Lyophilizer Alpha 1–2 LD plus, Martin Christ, Osterode, Germany).

### 3.3. Characterization

#### 3.3.1. Dynamic Light Scattering (DLS)

The hydrodynamic diameter $D_{H}$ of the particles was determined by dynamic light scattering using a Zetasizer Nano ZS (Malvern Instruments, Worcestershire, England) instrument. Re-dispersions of CUR, COU, and NOR were diluted with antisolvent and were measured with disposable polystyrene cuvettes (DTS0012, Malvern, Worcestershire, England). Re-dispersions of samples of AMI and PRO were measured undiluted in disposable microcuvettes (ZEN0040, Malvern, Worcestershire, England). The temperature was set to 25 °C, the scattering angle at 173°, and the viscosity at 0.8872 cP.

#### 3.3.2. Scanning Electron Microscopy (SEM)

Scanning electron microscopy (SUPRA 55-VP, Zeiss, Oberkochen, Germany) was used for evaluating the morphology of the particles and confirming the particle size using secondary electron detectors (inLens and SE). Samples prepared by the solvent-evaporation or freeze-drying of the compound particles were put on carbon tape and coated with iridium (PECS Model 680, Gatan, Pleasanton, CA, USA). For solvent-evaporation, droplets of compound re-dispersions were put on microscopy slides and allowed to dry at room temperature. Afterwards, these samples were coated with carbon (Turbo Carbon Coater, Agar, Essex, United Kingdom).

#### 3.3.3. Zeta Potential and pH Measurements

The zeta potential (ZP) of the re-dispersions (in antisolvent) of the particles of the end dispersions of protocol No. 1 was measured using the Zetasizer. The measurements were carried out at 25 °C with disposable folded capillary cells (DTS1070, Malvern, Worcestershire, United Kingdom) using the Zetasizer and Smoluchowski approximation. The value of pH was also measured with a 914 pH/Conductometer (Metrohm, Herisau, Switzerland) after calibration with buffers with pH = 4.008, pH = 7.413, and pH = 11.00.

#### 3.3.4. X-ray Powder Diffraction (XRD)

The crystal structures of freeze-dried compound particles were determined with a Bruker D8 Advance diffractometer (Cu Kα, λ = 1.5600 Å, Billerica, MA, USA) using a spinning flat plate configuration.

## 4. Results

### 4.1. Size Measurements

**Figure 1:**
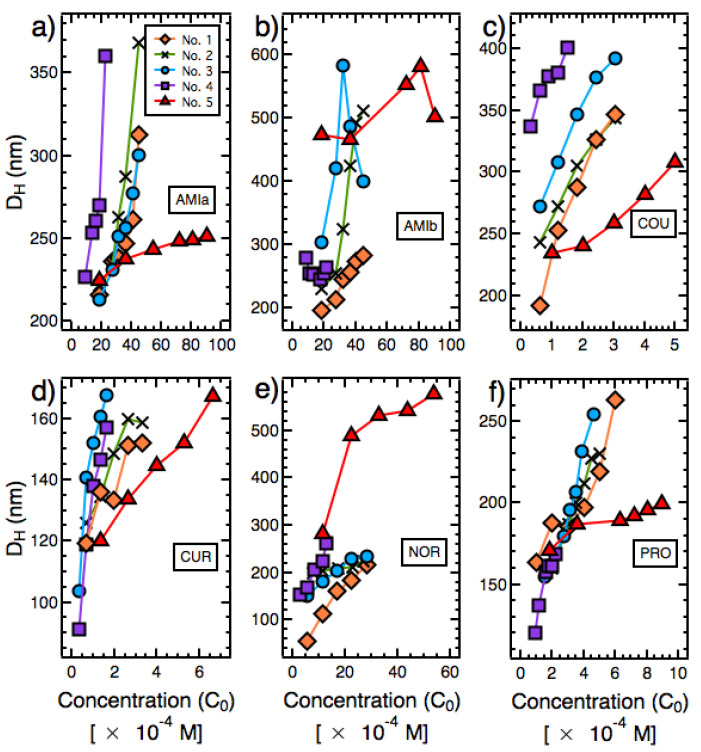
Mean particle size ($D_{H}$) of (**a**) AMIa, (**b**) AMIb, (**c**) COU, (**d**) CUR, (**e**) NOR, and (**f**) PRO with varying compound concentration in the solvent + antisolvent mixture solution ($C_{0}$).

For preparation protocols No. 1–No. 5 (Table 1), the results of the measurement of $D_{H}$ are presented in Figure 1 and Table 2. The ranges within which $D_{H}$ varied for preparation protocols No. 1–No. 5 were 216–369 nm (AMIa), 197–745 nm (AMIb), 193–401 nm (COU), 91–168 nm (CUR), 56–577 nm (NOR), and 120–264 nm (PRO) (Table 2). The value of $D_{H}$ increased with $C_{0},$ achieved through adding more solvent solution into the antisiolvent (Figure 1). For the majority of samples studied, the size distribution was unimodal (Supplementary Figure S1). Exceptions included (i) two instances where large particles (5000–6000 nm) with very low intensity were observed (Supplementary Figure S1a,) and (ii) one instance where a fraction of small particles with low intensity was detected (Supplementary Figure S1c).

### 4.2. Measurements of Zeta Potential and pH and Colloidal Stability

**Figure 2:**
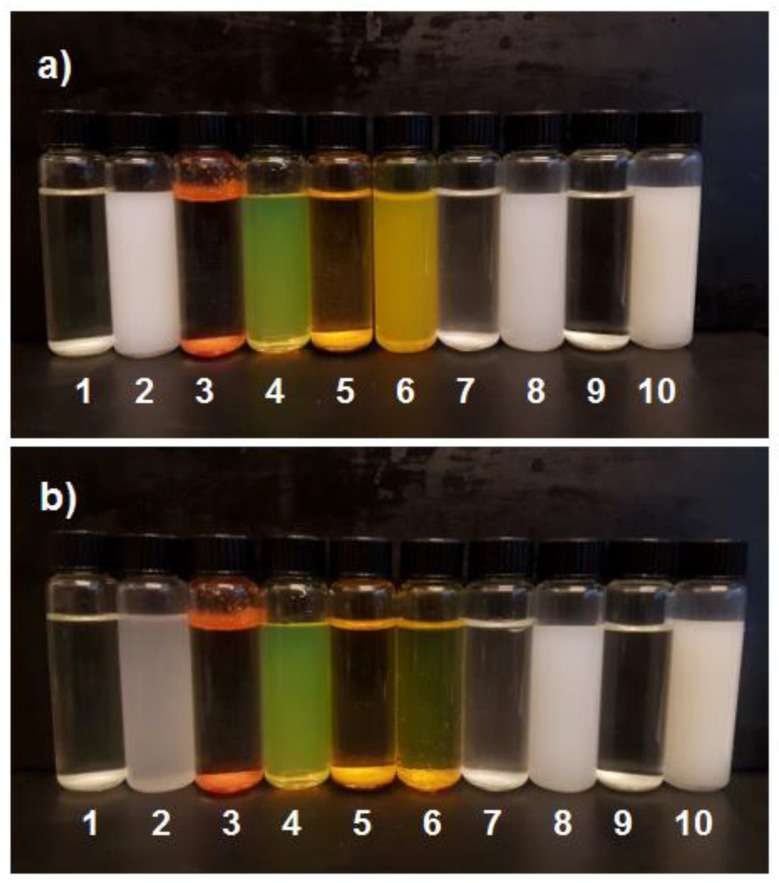
Visual inspection of the status of the dispersion for AMI (1 and 2), COU (3 and 4), CUR (5 and 6), NOR (7 and 8) and PRO (9 and 10) before (i.e., in the solvent solution) and after antisolvent precipitation, respectively, performed for (**a**) fresh samples and (**b**) after 7 days.

The results of the measurements of ZP and pH of the re-dispersions (in antisolvent) of the particles of the end dispersions of protocol No. 1 are presented in Table 3. Dispersions with ZP above +30 mV or below −30 mV are generally considered colloidally stable. CUR is relatively neutral with a low capability to protonate or deprotonate. Apparently, its weak basic properties bestowed it some degree of protonation at the studied pH (pH = 6.784 < pKa = 8.11), which resulted in a low positive ZP value (=1.0 mV; Table 3) for its particles. This low ZP value could not induce colloidal stability for the particles of CUR, because the surface charge-induced repulsive force between the particles was too low to keep them apart from each other. Indeed, after seven days, the only particles that precipitated in the solution were those of CUR (Figure 2). AMI and NOR are bases (see Table 3 for their pKa values), meaning that the positive ZP values observed for these molecules (Table 3) were due to their protonated states at the studied pH (pH < pKa). In the solution of AMI, compared to the solution of NOR, pH was further away from pKa (pKa-pH = 2.724 versus 0.395, respectively; see Table 3). This resulted in a higher ZP value for AMI particles (45.3 mV) compared to NOR particles (15.6 mV). Conversely, COU is an acid (pKa = 2.98), meaning that at the studied pH (= 7.203) its molecules were negatively charged, resulting in a negative ZP value (−22.8 mV) for its particles. PRO is a weak base (pKa = 7.66), and at the studied pH (= 12.426) its molecules were uncharged. Conversely, its co-crystal, maleic acid, should be deprotonated at the studied pH. Thus, the observation of a negative ZP value (−44.1 mV) for the particles of PRO was attributed to the adsorption of negatively charged maleate molecules by the surface of the particles.

### 4.3. Morphology

**Figure 3:**
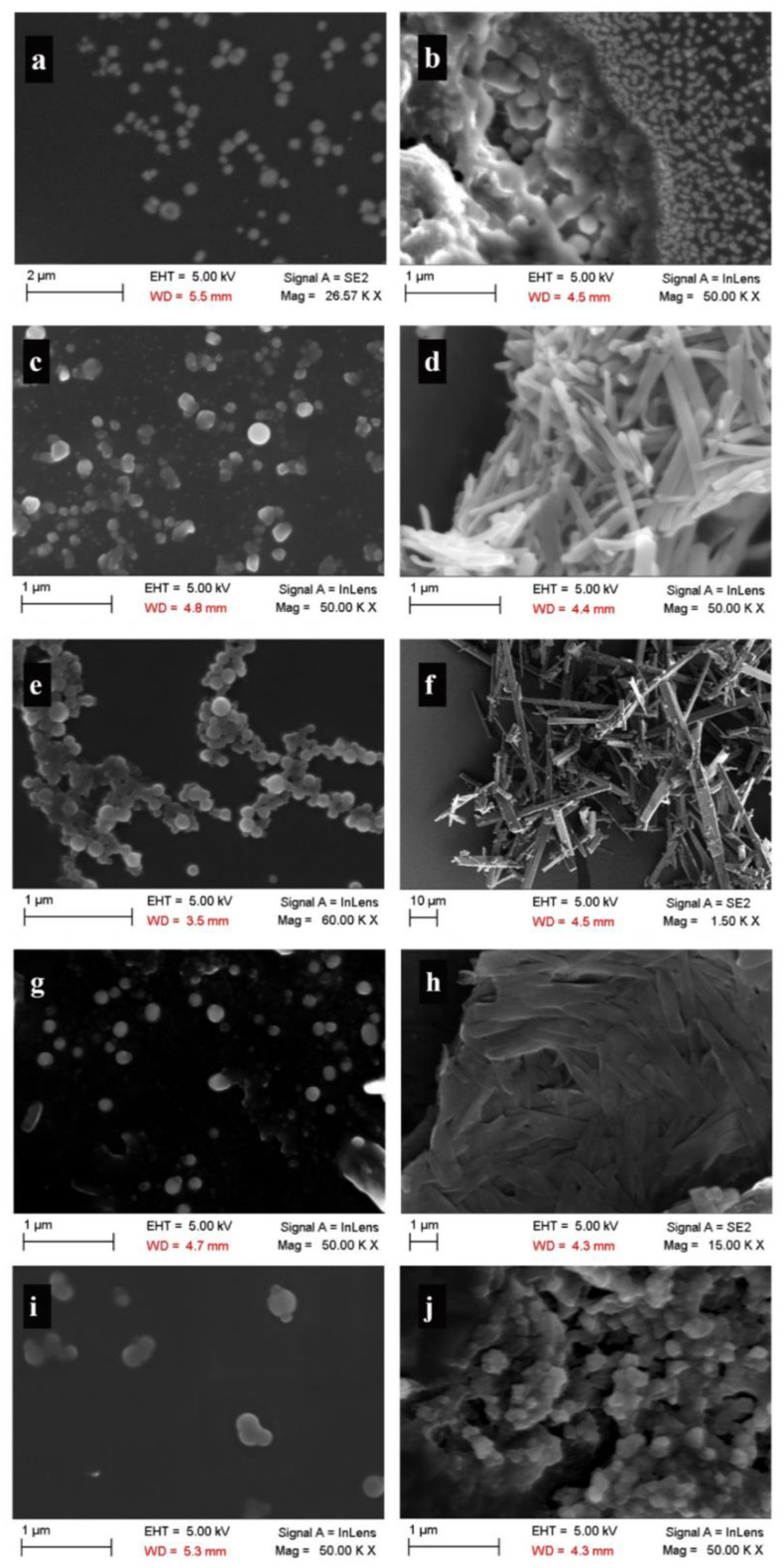
SEM images of particles of (**a**) AMIa, (**b**) AMIb, (**c**,**d**) COU, (**e**,**f**) CUR, (**g**,**h**) NOR, and (**i**,**j**) PRO generated by antisolvent precipitation method. (**a**,**c**,**e**,**g**,**i**) Solvent-evaporated particles. (**b**,**d**,**f**,**h**,**j**) Freeze-dried particles.

The morphology of the produced particles was observed by SEM. All of the particles that were dried by the evaporation of solvent were spherical in their shape (Figure 3), where the ranges of their sizes were similar to those found by DLS measurements. The freeze-dried particles showed two different morphologies. For AMI and PRO, the freeze-dried particles consisted of clusters of nanoparticles with sizes in the same range as that obtained before freeze-drying for solvent-evaporated samples. Freeze-drying induced a needle-like morphology for CUR, COU, and NOR particles, where for CUR and NOR the sizes of the particles became much larger than those obtained before freeze-drying for solvent-evaporated samples.

### 4.4. Analysis of XRD Results

**Figure 4:**
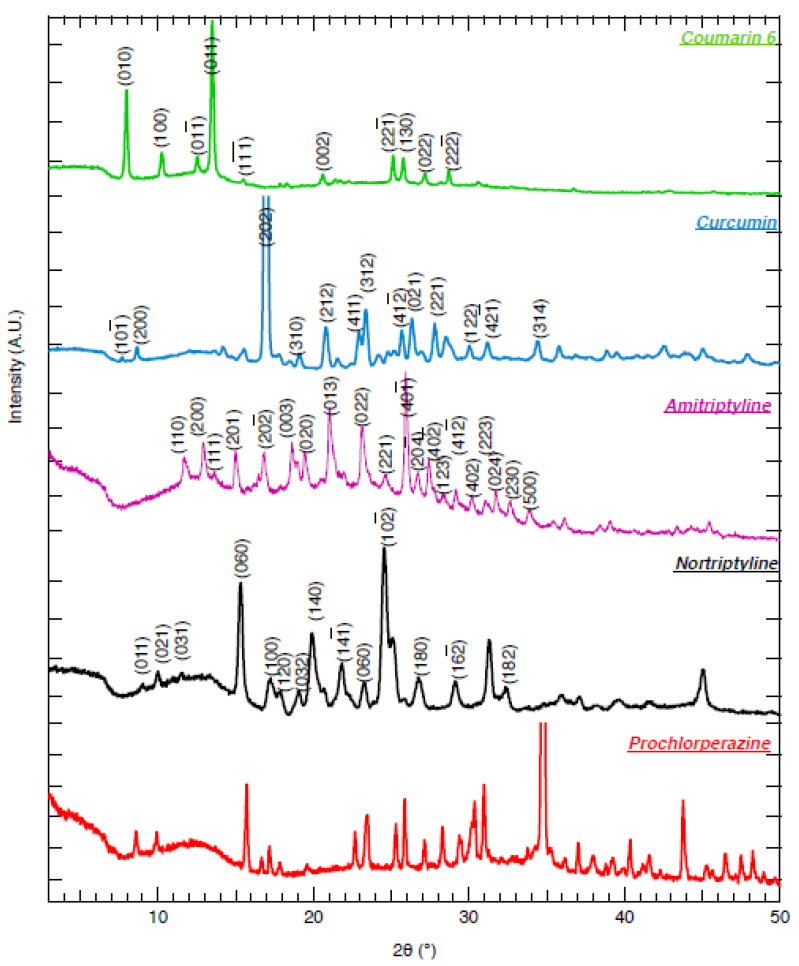
XRD data of freeze-dried samples of COU, CUR, AMI, NOR, and PRO particles. The XRD intensities are presented in logarithmic scales. The Miller indices of the main peaks are indicated for COU, CUR, AMI and NOR.

The X-ray powder diffraction (XRD) patterns for COU, CUR, AMI, NOR, and PRO can be found in Figure 4. The Cambridge Crystallographic Data Centre (CCDC) was used to identify and analyze the crystal unit cell and to index the peaks. The Miller indices for the main peaks of the first four compounds are presented in Figure 4. Prochlorperazine as a co-salt with methanesulphonic sulphonic acid was found to crystallize in monoclinic space group C2/c [43]. The single crystal structure for prochlorperazine as a co-salt with dimaleate was not found. Therefore, the peaks were not indexed for this sample.

Based on the XRD patterns, all of the compounds gave crystalline particles. CUR has been reported to crystallize in two different polymorph forms, namely form 1 and form 2 [44]. Obtained XRD data for CUR followed the pattern of polymorph form 1. NOR appeared in two polymorphic forms, namely form-α and form-β [45], but further characterization is necessary to completely distinguish the two forms, which is beyond the aim of the present study.

## 5. Discussion

### 5.1. General Remarks

The spherical shape of the particles was confirmed through inspection of the SEM images obtained for solvent-evaporated samples (Figure 2a,c,e,g,i). Thus, the assumption of a spherical shape for the particles made in the analysis of DLS data for obtaining $D_{H}$ is justified. The crystalline nature of the particles was confirmed through XRD analysis (Figure 4). Evidently, the colloidal stability of the end dispersions (Figure 3) correlated with ZP of the particles (Table 3; for details see Section 4.2).

Surface-active properties have been reported for AMI [25], NOR [25], and PRO [46]. For these compounds, a charged state of the molecule is the prerequisite for exhibiting surface-active properties. Since these compounds are weak bases, the percentage of the charged compound molecules (α%) can be estimated from the Hendersen–Hasselbach equation in the following manner.

(6) $$\alpha \%=\frac{100}{1+10^{\left(pH-pK_{a}\right)}}$$

Knowing the pH of the antisolvent (Table 1), the values of α% for the end dispersions of AMI, NOR, and PRO particles in the preparation process were estimated to be 9.68(±0.20)% (AMIa), 2.62(±0.06)% (AMIb), 9.68(±0.20)% (NOR), and 0.049(±0.002)% (PRO). This means that only AMI and NOR were charged enough to be identified as surface-active molecules during the experiments.

In the following, the effects of various parameters, including the flow rate, $C_{0}^{solvent}$, the SAS-ratio, and pH of the antisolvent solution, on $D_{H}^{f}$ will be discussed. Moreover, we will show that $D_{H}^{f}$ increases with ${\left(\log P\right)}_{av}$, where ${\left(D_{H}^{f}\right)}_{av}$ increases linearly with ${\left(\log P\right)}_{av}$.

### 5.2. Flow Rate

Flow rate usually has a minor impact on the particle size, because it exerts two opposing effects with respect to supersaturation [47,48,49,50]. (I) On the one hand, an increase in the flow rate is associated with an increased mixing efficiency, which results in a more uniform supersaturation through decreasing the local supersaturation, which induces a reduced size for the particles and a narrower particle size distribution [22,29]. (II) On the other hand, an increase in the flow rate increases the solvent composition in the solution, which increases $C^{*}$ and, consequently, decreases β, thus inducing an increase in the size of the particles.

**Figure 5:**
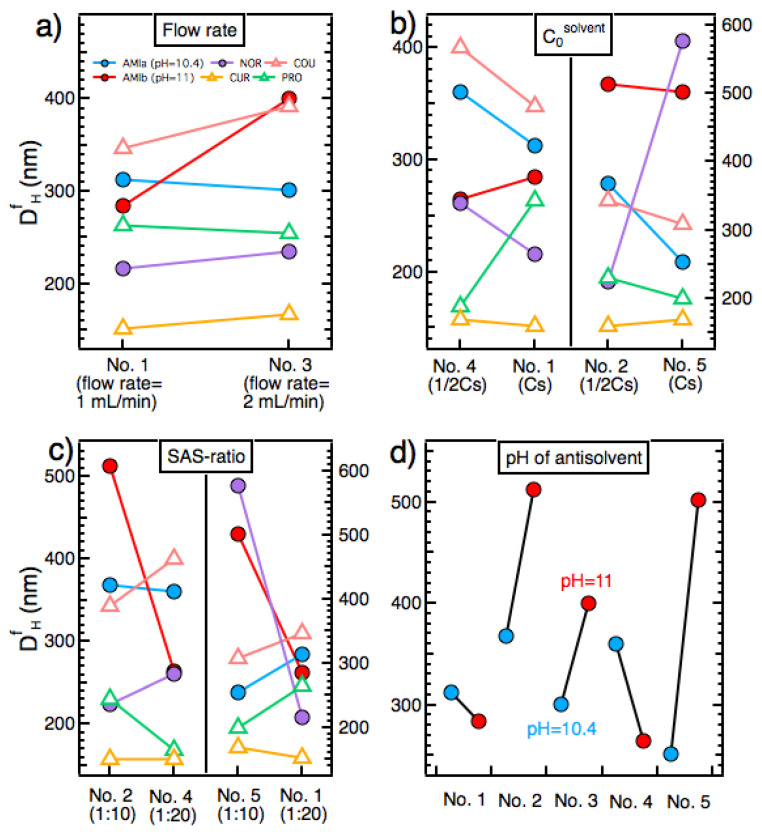
The effects of (**a**) flow rate, (**b**) compound concentration in the solvent solution $C_{0}^{solvent}$, (**c**) SAS-ratio (for AMIa, COU, CUR, NOR and PRO), and (**d**) pH of the antisolvent (for AMI particles prepared by protocols No.1–No.5; Table 2) on the final particle size $D_{H}^{f}$. All subfigures share the legend provided in (**a**).

Our results show that increasing the flow rate from 1 mL/min to 2 mL/min (protocols No. 1 and No. 3, respectively; Table 2) resulted in a narrower size distribution for NOR, AMIa, and AMIb (Supplementary Figure S1). A narrower size distribution for the particles has also been observed in other studies upon increasing the flow rate [22,29]. Moreover, $D_{H}^{f}$ of COU and AMIb increased largely once the flow rate was increased (Figure 5a). Conversely, for AMIa, NOR, CUR, and PRO, the changes in $D_{H}^{f}$ were subtle. Apparently, in the latter cases, the size-reducing effect explained in (I) and the size-enlarging effect described in (II) more or less cancelled each other.

### 5.3. Compound Concentration in the Solvent Solution ($C_{0}^{solvent}$)

In our experiments, increasing $C_{0}^{solvent}$ from ½Cs to Cs could induce the formation of smaller or larger particles (or particles with little change in their size) (Figure 5b). This occurred because a change in $C_{0}^{solvent}$ could influence the particle size in opposing ways (that sometimes may cancel each other), as explained in the following.

(I) On the one hand, a higher $C_{0}^{solvent}$ could induce formation of smaller particles, due to a lower $r^{*}$ (Equation (3)) and an enhanced J (Equation (4)) brought about by a higher β, because of a higher $C_{0}$ (Equation (1)). Nevertheless, it should be pointed out that the decrease in the particle size induced by an increased J has a minimum size limit, because J itself approaches an asymptotic maximum upon increasing β (Equation (4)). This means that once this minimum size limit is achieved, a further increase in β (e.g., through increasing $C_{0}^{solvent}$) does not affect the particle size through influencing J [51].

(II) On the other hand, a higher $C_{0}^{solvent}$ could induce the formation of larger particles, because it accelerates the adhesion and agglomeration of particles by increasing $r_{s}$. More specifically, a higher $C_{0}^{solvent}$ could induce the formation of a larger number of nuclei due to a to a lower $r^{*}$ (Equation (3)) and an enhanced J (Equation (4)) (see the discussion above in (I)), which results in a larger $n_{p}$, and thereby a higher $r_{s}$ as described by the Smoluchowski kinetics rate (Equation (5)).

### 5.4. Solvent: Antisolvent Ratio (SAS-Ratio)

In our experiments, increasing the SAS-ratio from 1:10 to 1:20 could induce the formation of smaller or larger particles (or particles with little change in their size) (Figure 5c). This happened because a change in SAS-ratio could influence the particle size in opposing ways (that sometimes may cancel each other), as explained in the following. (I) On the one hand, a higher SAS-ratio could induce the formation of smaller particles, due to a lower $r^{*}$ (Equation (3)) and an enhanced J (Equation (4)) brought about by a higher β, because of a smaller $C^{*}$ (Equation (1)). (II) On the other hand, a higher SAS-ratio could induce the formation of larger particles, due to a higher γ, which would induce a higher $r^{*}$ (Equation (3)) and a lower J (Equation (4)).

### 5.5. Surface Active Compounds

Sometimes, changing a process parameter could induce a very large change in $D_{H}^{f}$ of AMI and NOR, which are surface-active compounds (Figure 5a–c). In particular, very large increases were noted in one instance for $D_{H}^{f}$ of NOR upon increasing $C_{0}^{solvent}$ (Figure 5b), and three instances for NOR and AMIb upon decreasing the SAS-ratio (Figure 5c). For AMIb and NOR, the value of α% was low (α%<3%; see subSection 5.1), meaning that an aggregation/adhesion tendency for the particles was not unexpected, because the stabilizing electrostatic repulsive force between the particles was low. Examination of the data of Figure 1b,e revealed that in these instances, the rate of increase in $D_{H}$ in the protocols employed (No. 2 and No. 5 for AMIb and No. 5 for NOR) was very large at the early stage of the process of adding the solvent into the antisolvent (i.e., a jump was observed in $D_{H}$), whereas in the protocols that were used for comparison with these instances (No. 1 and No. 4 for AMIb and No. 1 and No. 2 for NOR), the rate of increase in $D_{H}^{f}$ was more or less constant during the whole process. Thus, we suggest that in these instances of very large increases in $D_{H}^{f}$, an enhanced aggregation/adhesion tendency was in play during the early stage of the process of adding the solvent into the antisolvent.

### 5.6. pH of the Antisolvent

For studying the effect of pH of the antisolvent on the particle size, we selected AMI (Figure 5d), where two pH values of pH = 10.4 (AMIa) and pH = 11 (AMIb) were chosen for the experiments (Table 1). Lowering pH from 11 to 10.4 affected $D_{H}^{f}$ of AMI in opposing manners via increasing α% from 2.62 (±0.06)% to 9.68 (±0.2)%. (I) On the one hand, an increased α% could result in smaller particles through a reduced γ and/or a decreased aggregation/adhesion tendency, where the latter is achieved through a larger degree of stabilizing electrostatic repulsion between the particles. (II) On the other hand, an increased α% could induce the formation of larger particles through a reduced β brought about by a higher AMI solubility ($C^{*}$; Equation (1)) [31]. In the following, the underlying physical chemistry of these two tendencies will be discussed in detail.

For bases (B) that are much less soluble in water than their corresponding salts (e.g., amitriptyline hydrochloride [31]), the relationship between the water solubility $C_{B}^{*,w}$ and pH is described by the following expressions [31].

(7) $$C_{B}^{*,w}=C_{f.b.}^{*,w}\left(1+\frac{\left[H^{+}\right]}{K_{a}}\right)$$

(8) $$K_{a}=\frac{\left[H^{+}\right]\left[B\right]}{\left[HB^{+}\right]}$$

where $C_{f.b.}^{*,w}$ is the water solubility of the free base of compound B and $K_{a}$ is the ionization constant. Thus, using the values of AMI free base water solubility ($C_{f.b.}^{*,w}=3.5\times 10^{-5}\;M$) and ionization constant ($pK_{a}=9.4$) (measured at 24 ± 1 °C [31]), the values of the water solubility of AMI in the end dispersion at different pH values of the antisolvent were calculated to be $C_{AMI}^{*,w}=3.6\times 10^{-5}$ M (pH = 11) and $C_{AMI}^{*,w}=3.9\times 10^{-5}$ M (pH = 10.4). This change in $C_{AMI}^{*,w}$ affects β through changing $C^{*}$ (Equation (1)). More specifically, lowering the pH of the antisolvent decreases β, which could increase $D_{H}^{f}$ through inducing a larger $r^{*}$ (Equation (3)) and a lower J (Equation (4)).

A change in the water solubility of AMI also affects γ. AMI is a surface-active molecule that self-assembles into micelles in water [25]. The critical micelle concentration (CMC) of this compound is CMC = 3.6 × 10^−2^ M and its aggregation number is 7 (measured at 303 ± 0.01 K [25]). The surface-active properties of AMI are pH-dependent, because it is a weak base. Add to this is the fact that its CMC is three orders of magnitude larger than the water solubility of its free base. This means that the contribution of the free base to CMC is negligible, and the occurrence of CMC is due to the presence of the ionized form of AMI. Besides, it is a well-known characteristic of aqueous solutions of surfactants that the surface tension sharply decreases with the surfactant concentration if the latter is less than CMC. This behavior continues up to CMC, after which the surface tension achieves a plateau value. Thus, because at pH = 11 and pH = 10.4 the water solubility of AMI is much smaller than CMC (i.e., $C_{AMI}^{*,w}\ll \mathrm{CMC}$), lowering the pH from 11 to 10.4 reduces γ, which results in a decrease in $D_{H}^{f}$ through inducing a smaller $r^{*}$ (Equation (3)) and a higher J (Equation (4)).

Inspection of the data of Figure 5d reveals that in preparation protocols No. 2, No. 3, and No. 5, lowering the pH from pH = 11 to pH = 10.4 induced a decrease in $D_{H}^{f}$. This means that, in these experiments, upon a decrease in pH, the size-reducing effect arising from a reduced aggregation/adhesion tendency and/or a reduced γ outweighed that of the size-enlarging effect of a reduced β. Conversely, in protocols No. 1 and No. 4, it was the size-enlarging effect of a reduced β that dominated the system, which resulted in an increased $D_{H}^{f}$ for a lower pH value. Thus, the fact that a small change in pH (ΔpH = 0.6) could exert such a great influence on $D_{H}^{f}$ of AMI particles is directly related to the surface-active properties of this compound.

### 5.7. ${\left(logP\right)}_{av}$

**Figure 6:**
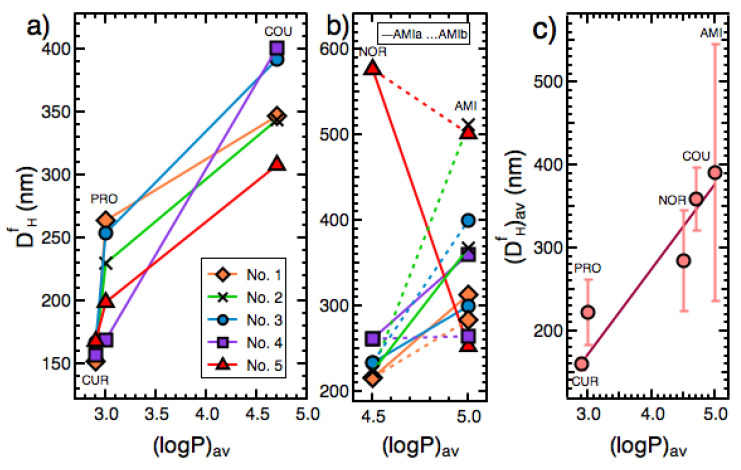
For CUR, PRO, COU, AMI and NOR, the relationship between the average of logP values for each compound ${\left(\log P\right)}_{av}$ (Table 3) and (**a**,**b**) the final particle size $D_{H}^{f}$ for various particle preparation protocols, and (c) the average of the values of the final particle size ${\left(D_{H}^{f}\right)}_{av}$. In (c), the solid line is the result of fitting a line to the data (R^2^ = 0.52).

For COU, CUR, and PRO particles, the concentration of the compound in the solvent solution varied in the range $3\;mM<C_{0}^{solvent}<10\;mM$ (Table 1). This means that $C_{0}^{solvent}$ was of the same order of magnitude in the preparation experiments. Interestingly, we found that for all of the preparation protocols applied for COU, CUR, and PRO, $D_{H}^{f}$ increased with ${\left(\log P\right)}_{av}$ (Figure 6a). We suggest that this happened, because a higher ${\left(\log P\right)}_{av}$ translated into a higher γ, and thereby a larger $D_{H}^{f}$, achieved through a larger $r^{*}$ (Equation (3)) and a lower J (Equation (4)).

As discussed before in Section 5.1, in the employed preparation protocols, AMI and NOR were expected to be charged enough to be identified as surface-active molecules. In addition, the concentration of these compounds in the solvent solution varied in the range $25\;\mathrm{mM}\leq C_{0}^{solvent}<80\;\mathrm{mM}$ (Table 1), which was one order of magnitude larger than the case for COU, CUR, and PRO. For these two reasons, analysis of the relationship between ${\left(\log P\right)}_{av}$ and $D_{H}^{f}$ for AMI and NOR for each preparation protocol cannot be included within that performed for COU, CUR, and PRO and should be carried out separately. Thus, upon comparing $D_{H}^{f}$ of AMIa, AMIb, and NOR, the same conclusion regarding the relationship between ${\left(\log P\right)}_{av}$ and $D_{H}^{f}$ was reached, meaning that in the majority of the preparation protocols employed, the compound with a lower ${\left(\log P\right)}_{av}$ (i.e, NOR) exhibited a smaller $D_{H}^{f}$ (Figure 6b). Furthermore, as can be seen in Figure 6c, ${\left(D_{H}^{f}\right)}_{av}$ increased linearly with ${\left(\log P\right)}_{av}$ (${\left(D_{H}^{f}\right)}_{av}$ was calculated by making an average of $D_{H}^{f}$ values obtained for the five preparation protocols employed. Notice that for the calculation of ${\left(D_{H}^{f}\right)}_{av}$ of AMI, both AMIa and AMIb particles were taken into account). These findings regarding the relationship between the final particle size and logP could be used as a general principle to predict the particle size in the preparation of nanodrugs using the antisolvent precipitation technique.

## 6. Conclusions

The antisolvent precipitation method was successfully employed in the preparation of excipient-free, pure nanoparticles of amitriptyline hydrochloride (AMI), coumarin 6 (COU), curcumin (CUR), nortriptyline hydrochloride (NOR), and prochlorperazine dimaleate (PRO) using five different preparation protocols (Table 2). The final size of the particles $D_{H}^{f}$ varied in the ranges of 245–745 nm (AMI), 308–401 nm (COU), 152–168 nm (CUR), 216–577 nm (NOR), and 169–264 nm (PRO). The effects of varying the flow rate, $C_{0}^{solvent}$, SAS-ratio, and pH of the antisolvent on $D_{H}^{f}$ were examined in detail and the results were explained using available theories for the antisolvent precipitation method. The morphology of the particles, their crystalline structure, and their surface charge were characterized using SEM, XRD, and zeta potential measurements, respectively. Importantly, we showed that $D_{H}^{f}$ increased with ${\left(\log P\right)}_{av}$, where ${\left(D_{H}^{f}\right)}_{av}$ increased linearly with ${\left(\log P\right)}_{av}$.

## Figures and Tables

**Table 1:** Compound concentration in the solvent solution ($C_{0}^{solvent}$; mM), centrifugation time (min), and the solvent and antisolvent used for each of the compounds investigated in the present study.

Compound	$C_{0}^{solvent}\;(\mathrm{mM})$	Centrifuging Time	Solvent	Antisolvent
AMIa	39.8–79.7	10 min × 2	Ethanol	0.25 mM NaOH (pH =10.4)
AMIb	39.8–79.7	10 min × 2	Ethanol	1.0 mM NaOH (pH = 11)
COU	3.14–6.28	10 min × 2	Ethanol	Distilled water
CUR	3.39–6.79	10 min × 2	Ethanol	Distilled water
NOR	25.0–50.0	10 min × 3	Ethanol	1.0 mM NaOH (pH = 11)
PRO	4.54–9.07	10 min × 2	Acetone, NaOH	1.0 mM NaOH (pH = 11)

**Table 2:** The parameters of the preparation protocols, such as the flow rate (mL/min), the compound concentration in the solvent solution ($C_{0}^{solvent}$) and the solvent:antisolvent ratio (SAS-ratio) together with the range of the particle size ($D_{H}$) are given. If the antisolvent is other than distilled water, its pH is indicated.

Preparation Protocol	Process Parameters			$\mathrm{Range}\;\mathrm{of}\;\mathrm{the}\;\mathrm{Particle}\;\mathrm{Size}\;(D_{H})\;(\mathrm{nm})$					
	Flow Rate	$C_{0}^{solvent}$	SAS-Ratio	AMIa (pH = 10.4)	AMIb (pH = 11)	COU	CUR	NOR (pH = 11)	PRO (pH = 11)
No. 1	1	Cs	1:20	216–313	197–284	193–347	119–152	56–216	163–264
No. 2	1	½ Cs	1:10	222–369	231–513	243–344	126–159	200–224	160–230
No. 3	2	Cs	1:20	213–301	306–745	273–392	104–168	151–235	155–255
No. 4	1	½ Cs	1:20	227–361	246–265	337–401	91–157	152–261	120–169
No. 5	1	Cs	1:10	225–254	467–582	235–308	120–168	282–577	171–199

**Table 3:** The results of the zeta potential and pH measurements of the re-dispersions (in antisolvent) of the particles of the end dispersions of protocol No. 1 together with the values of $pK_{a}$, logP and ${\left(\log P\right)}_{av}$ of the compounds.

Compound	ZP ± SD (mV)	pH ± SD	$pK_{a}$	logP	${\left(\log P\right)}_{av}$
AMI	45.3 ± 6.9	6.676 ± 0.071	9.4 ^a^	5.84 ^b^, 4.97 ^c^, 4.54 ^d^, 4.86 ^e^, 4.7 ^f^	5.0
COU	−22.8 ± 0.3	7.203 ± 0.042	2.98 ^g^	4.89 ^b^, 4.92 ^c^, 3.47 ^d^, 5.48 ^e^	4.7
CUR	1.0 ± 0.1	6.784 ± 0.078	8.11 ^h^	3.20 ^b^, 3.15 ^c^, 1.47 ^d^, 4.04 ^e^, 2.5 ^i^	2.9
NOR	15.6 ± 0.7	9.605 ± 0.019	10.0 ^j^	5.31 ^b^, 4.63 ^c^, 4.31 ^d^, 4.92 ^e^, 3.57 ^f^	4.5
PRO	−44.1 ± 4.4	12.426 ± 0.026	7.66 ^k^	2.86 ^c^, 2.21 ^d^, 3.85 ^e^, 3 ^f^	3.0

^a^ [31]; ^b^ [32,33]; ^c^ [33,34]; ^d^ [33,35,36]; ^e^ [33]; ^f^ [37]; ^g^ [38]; ^h^ [39]; ^i^ [40]; ^j^ [41]; ^k^ [42].

## Data Availability

Not applicable.

## Supplementary Materials

The following supporting information can be downloaded at: https://www.mdpi.com/article/10.3390/pharmaceutics14040819/s1, Figure S1: Representative size distributions of particles of (a) CUR, (b) COU, (c) NOR, (d) PRO, (e) AMIa and (f) AMIb. The preparation protocols employed are indicated.

## References

[1] Semahegn A., Torpey K., Manu A., Assefa N., Tesfaye G., Ankomah A. (2018). Psychotropic medication non-adherence and associated factors among adult patients with major psychiatric disorders: A protocol for a systematic review. Syst. Rev..

[2] Roy R., Jahan M., Kumari S. (2005). Reasons for Drug Non-Compliance of Psychiatric Patients: A Centre Based Study. J. Indian Acad. Appl. Psychol..

[3] Ng Q.X., Koh S.S.H., Chan H.W., Ho C.Y.X. (2017). Clinical Use of Curcumin in Depression: A Meta-Analysis. J. Am. Med. Dir. Assoc..

[4] Peltonen L., Hirvonen J. (2018). Drug nanocrystals—Versatile option for formulation of poorly soluble materials. Int. J. Pharm..

[5] Song S., Gui L., Feng Q., Taledaohan A., Li Y., Wang W., Wang Y., Wang Y. (2020). TAT-Modified Gold Nanoparticles Enhance the Antitumor Activity of PAD4 Inhibitors. Int. J. Nanomed..

[6] Abbott N.J., Patabendige A.A.K., Dolman D.E.M., Yusof S.R., Begley D.J. (2010). Structure and function of the blood-brain barrier. Neurobiol. Dis..

[7] Fowler M.J., Cotter J.D., Knight B.E., Sevick-Muraca E.M., Sandberg D.I., Sirianni R.W. (2020). Intrathecal drug delivery in the era of nanomedicine. Adv. Drug Deliv. Rev..

[8] Lesniak W.G., Chu C., Jablonska A., Azad B.B., Zwaenepoel O., Zawadzki M., Lisok A., Pomper M.G., Walczak P., Gettemans J. (2019). PET imaging of distinct brain uptake of a nanobody and similarly-sized PAMAM dendrimers after intra-arterial administration. Eur. J. Pediatr..

[9] Ye D., Chen H., Rasooly A., Baker H., Ossandon M.R. (2022). Focused Ultrasound—Mediated Intranasal Brain Drug Delivery Technique (FUSIN). Biomedical Engineering Technologies.

[10] Hersh D.S., Anastasiadis P., Mohammadabadi A., Nguyen B.A., Guo S., Winkles J.A., Kim A.J., Gullapalli R., Keller A., Frenkel V. (2018). MR-guided transcranial focused ultrasound safely enhances interstitial dispersion of large polymeric nanoparticles in the living brain. PLoS ONE.

[11] Ventola C.L. (2017). Progress in nanomedicine: Approved and investigational nanodrugs. Pharm. Ther..

[12] Verma S., Gokhale R., Burgess D.J. (2009). A comparative study of top-down and bottom-up approaches for the preparation of micro/nanosuspensions. Int. J. Pharm..

[13] Rabinow B.E. (2004). Nanosuspensions in drug delivery. Nat. Rev. Drug Discov..

[14] Chan H.-K., Kwok P.C.L. (2011). Production methods for nanodrug particles using the bottom-up approach. Adv. Drug Deliv. Rev..

[15] Le Bourlais C., Acar L., Zia H., Sado P.A., Needham T., Leverge R. (1998). Ophthalmic drug delivery systems—Recent advances. Prog. Retin. Eye Res..

[16] Loh Z.H., Samanta A.K., Heng P.W.S. (2015). Overview of milling techniques for improving the solubility of poorly water-soluble drugs. Asian J. Pharm. Sci..

[17] Yadav D., Kumar N. (2014). Nanonization of curcumin by antisolvent precipitation: Process development, characterization, freeze drying and stability performance. Int. J. Pharm..

[18] Patel R., Baria A., Patel N. (2008). An overview of size reduction technologies in the field of pharmaceutical manufacturing. Asian J. Pharm..

[19] Kakran M., Sahoo N.G., Tan I.-L., Li L. (2012). Preparation of nanoparticles of poorly water-soluble antioxidant curcumin by antisolvent precipitation methods. J. Nanoparticle Res..

[20] Schmid K., Arpagaus C., Friess W. (2010). Evaluation of the Nano Spray Dryer B-90 for pharmaceutical applications. Pharm. Dev. Technol..

[21] Zhao X., Zhao H., Wang S., Fan Z., Ma Y., Yin Y., Wang W., Xi R., Meng M. (2021). A Tumor-Targeting Near-Infrared Heptamethine Cyanine Photosensitizer with Twisted Molecular Structure for Enhanced Imaging-Guided Cancer Phototherapy. J. Am. Chem. Soc..

[22] Sinha B., Müller R.H., Möschwitzer J.P. (2013). Bottom-up approaches for preparing drug nanocrystals: Formulations and factors affecting particle size. Int. J. Pharm..

[23] Chen Y., Wang S., Xu X., Liu X., Yu M., Zhao S., Liu S., Qiu Y., Zhang T., Liu B.-F. (2013). Synthesis and Biological Investigation of Coumarin Piperazine (Piperidine) Derivatives as Potential Multireceptor Atypical Antipsychotics. J. Med. Chem..

[24] Chen Y., Lan Y., Wang S., Zhang H., Xu X., Liu X., Yu M., Liu B.-F., Zhang G. (2014). Synthesis and evaluation of new coumarin derivatives as potential atypical antipsychotics. Eur. J. Med. Chem..

[25] Attwood D., Gibson J. (2011). Aggregation of antidepressant drugs in aqueous solution. J. Pharm. Pharmacol..

[26] Söhnel O. (1993). Precipitation: Basic principles and Industrial Applications.

[27] Karthika S., Radhakrishnan T., Kalaichelvi P. (2016). A Review of Classical and Nonclassical Nucleation Theories. Cryst. Growth Des..

[28] Jungblut S., Dellago C. (2016). Pathways to self-organization: Crystallization via nucleation and growth. Eur. Phys. J. E.

[29] Dalvi S.V., Dave R.N. (2009). Controlling Particle Size of a Poorly Water-Soluble Drug Using Ultrasound and Stabilizers in Antisolvent Precipitation. Ind. Eng. Chem. Res..

[30] Abdelwahed W., Degobert G., Stainmesse S., Fessi H. (2006). Freeze-drying of nanoparticles: Formulation, process and storage considerations. Adv. Drug Deliv. Rev..

[31] Green A.L. (2011). Ionization constants and water solubilities of some aminoalkylphenothiazine tranquillizers and related compounds. J. Pharm. Pharmacol..

[32] Cheng T., Zhao Y., Li X., Lin F., Xu Y., Zhang X., Li A.Y., Wang R., Lai L. (2007). Computation of Octanol−Water Partition Coefficients by Guiding an Additive Model with Knowledge. J. Chem. Inf. Model..

[33] Daina A., Michielin O., Zoete V. (2017). SwissADME: A free web tool to evaluate pharmacokinetics, drug-likeness and medicinal chemistry friendliness of small molecules. Sci. Rep..

[34] Wildman S.A., Crippen G.M. (1999). Prediction of Physicochemical Parameters by Atomic Contributions. J. Chem. Inf. Comput. Sci..

[35] Moriguchi I., Hirono S., Nakagome I., Hirano H. (1994). Comparison of Reliability of log P Values for Drugs Calculated by Several Methods. Chem. Pharm. Bull..

[36] Moriguchi I., Hirono S., Liu Q., Nakagome I., Matsushita Y. (1992). Simple Method of Calculating Octanol/Water Partition Coefficient. Chem. Pharm. Bull..

[37] Frisk-Holmberg M., van der Kleijn E. (1972). The relationship between the lipophilic nature of tricyclic neuroleptics and antidepressants, and histamine release. Eur. J. Pharmacol..

[38] (2019). Coumarin 6, CAS=38215-36-0. https://scifinder-n.cas.org/searchDetail/substance/624ff3459b3b45668680912c/substanceDetails.

[39] (2019). Curcumin, CAS=458-37-7. https://scifinder-n.cas.org/searchDetail/substance/624ff3769b3b4566868091ba/substanceDetails.

[40] Priyadarsini K.I. (2009). Photophysics, photochemistry and photobiology of curcumin: Studies from organic solutions, bio-mimetics and living cells. J. Photochem. Photobiol. C Photochem. Rev..

[41] (2019). Nortriptyline Hydrochloride, CAS=894-71-3. https://scifinder-n.cas.org/searchDetail/substance/624ff3979b3b45668680921e/substanceDetails.

[42] (2019). Prochlorperazine Dimaleate, CAS=84-02-6. https://scifinder-n.cas.org/searchDetail/substance/624ff42a9b3b4566868093b5/substanceDetails.

[43] McDowell J.J.H. (1979). Prochlorperazine–methanesulphonic acid (1:2), a phenothiazine derivative. Acta Crystallogr. Sect. B Struct. Crystallogr. Cryst. Chem..

[44] Sanphui P., Goud N.R., Khandavilli U.B.R., Bhanoth S., Nangia A. (2011). New polymorphs of curcumin. Chem. Commun..

[45] Vladiskovic C., Masciocchi N., Cervellino A. (2012). A Structural Powder Diffraction Study of Two Polymorphic Forms of Nortriptyline Hydrochloride. J. Pharm. Sci..

[46] Seeman P.M., Bialy H.S. (1963). The surface activity of tranquilizers. Biochem. Pharmacol..

[47] Matos R.L., Lu T., Prosapio V., McConville C., Leeke G., Ingram A. (2019). Coprecipitation of curcumin/PVP with enhanced dissolution properties by the supercritical antisolvent process. J. CO2 Util..

[48] Martín Á., Cocero M. (2004). Numerical modeling of jet hydrodynamics, mass transfer, and crystallization kinetics in the supercritical antisolvent (SAS) process. J. Supercrit. Fluids.

[49] Erriguible A., Fadli T., Subra-Paternault P. (2013). A complete 3D simulation of a crystallization process induced by supercritical CO_2_ to predict particle size. Comput. Chem. Eng..

[50] Lengsfeld C.S., Delplanque J.P., Barocas V.H., Randolph T.W. (2000). Mechanism Governing Microparticle Morphology during Precipitation by a Compressed Antisolvent: Atomization vs Nucleation and Growth. J. Phys. Chem. B.

[51] Wang Z., Chen J.-F., Le A.Y., Shen Z.-G., Yun J. (2007). Preparation of Ultrafine Beclomethasone Dipropionate Drug Powder by Antisolvent Precipitation. Ind. Eng. Chem. Res..

