# Supplementary material for: Application of Antisolvent Precipitation Method for Formulating Excipient-Free Nanoparticles of Psychotropic Drugs
Source: Pharmaceutics. 2022 Apr 8;14(4):819. doi: 10.3390/pharmaceutics14040819 (PMC9029518; doi:10.3390/pharmaceutics14040819)
Supplement: Supplementary file 1 [file pharmaceutics-14-00819-s001.zip › pharmaceutics-1655146-supplementary.pdf]

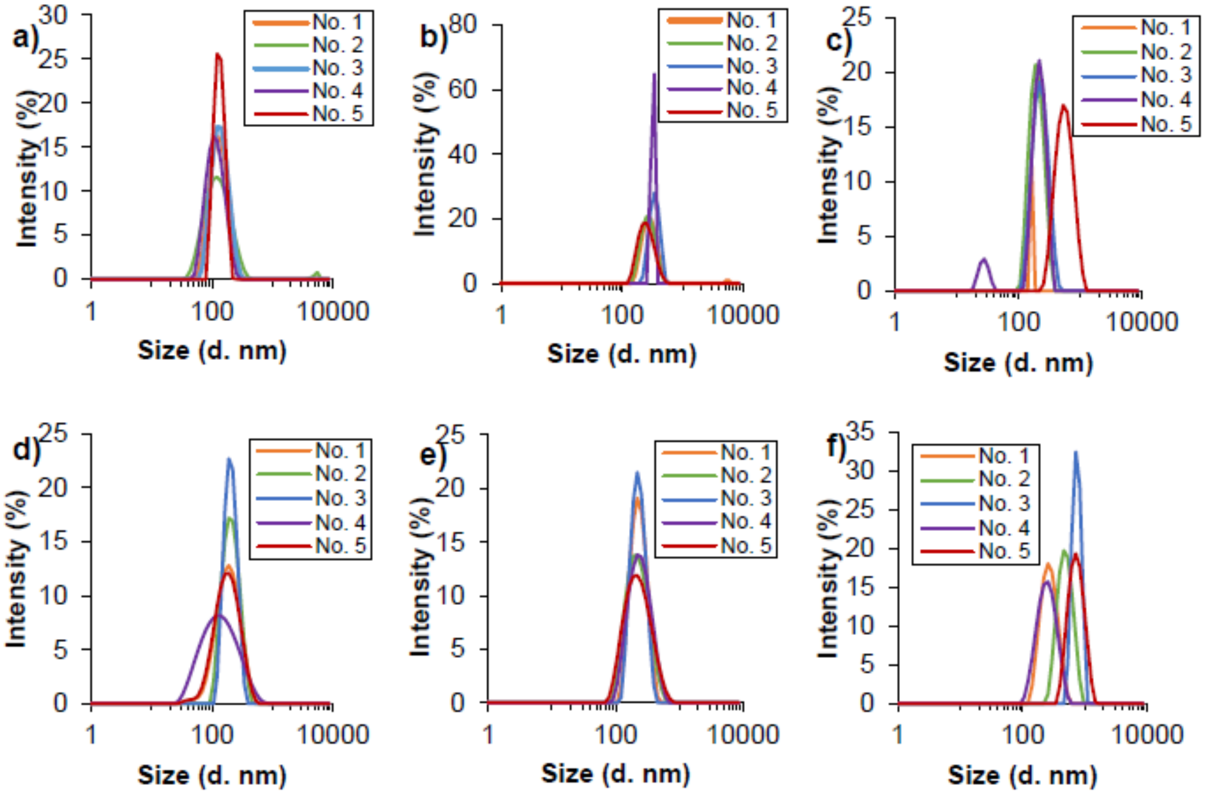

Figure S1. Representative size distributions of particles of (a) CUR, (b) COU, (c) NOR, (d) PRO, (e) AMIa and (f) AMIb. The preparation protocols employed are indicated.
